# Supplementary material for: Using control charts to understand community variation in COVID-19
Source: PLoS One. 2021 Apr 30;16(4):e0248500. doi: 10.1371/journal.pone.0248500 (PMC8087083; doi:10.1371/journal.pone.0248500)
Supplement: Attachment — Submitted filename: Response to Reviewers v2.docx [file pone.0248500.s002.docx]

Thank you for the helpful review. We addressed each comment in the revised narrative. Our responses to the reviews are provided below.

1. Results, page 12. The results state that household crowding at Westlake occurred in 45% of households, but the revised Table 1 lists 45% for Westlake household crowding. The results also describe Westlake as having a large household size with median size of 4.0; however, Table 1 gives median household size of 3.0 for Westlake, which is the second smallest household size among the five cities/neighborhoods studied. Could you please clarify this apparent discrepancy and ensure that all table/manuscript statistics are accurate?

*Response:* We corrected a typographical error in the narrative that resulted in a discrepancy between the narrative and Table 1. We deleted a sentence that had some redundant information. We reviewed the data in the narrative, and in Table 1, and all other data are correct.

2. Results, page 12. I recommend removing the last paragraph that mentions publicly available stratification of counts. This comment is useful but is better suited for the discussion section. This comment could be folded into the related discussion of general public vs. congregate living populations on page 14.

Response: As suggested, we deleted this last paragraph from Results and added this point into the Discussion (Page 15) as follows: *“Local jurisdictions may need this differentiation to target strategies – for example, if most cases are in congregate facilities.”*

3. Discussion/Conclusions sections. I appreciate the revised and improved discussion and conclusions sections. Additional copyediting of these sections by the authors would improve readability and impact. While these changes would be stylistic and overall minor, I think editing by the authors at this stage is important because the journal does not provide copyediting.

Response: We appreciate the suggestion of additional copy editing. We combined two paragraphs in the Discussion and made additional line edits to improve the clarity of this section. The changes are tracked in the revised narrative. Additionally, we addressed the specific copyediting suggestions (please see below).

Here are some examples of the type of copyediting that may improve readability of these sections:

a) Page 13, line 3. Could consider: “...tool for daily learning and action that could benefit public health.”

*Response:* We made this edit in the narrative.

b) Page 13, last sentence of paragraph 1. Could consider: “Use of control charts could reduce overreaction to expected random variation and encourage immediate action when…”

*Response:* We made this change.

c) Page 14, second paragraph. Could consider: “Data alone do not tell the full story.”

*Response:* We deleted this sentence and instead edited the next sentence to read: *“Chart annotation makes it possible to…”*

d) Page 17. Could consider: “Use of control charts in public health will require an appreciation of their value and investment of time and resources into their creation and use.”

*Response:* We made this edit in the revised narrative.
